# Spike Gene Target Amplification in a Diagnostic Assay as a Marker for Public Health Monitoring of Emerging SARS-CoV-2 Variants — United States, November 2021–January 2023

**DOI:** 10.15585/mmwr.mm7205e2

**Published:** 2023-02-03

**Authors:** Heather M. Scobie, Akilah R. Ali, Philip Shirk, Zachary R. Smith, Prabasaj Paul, Clinton R. Paden, Norman Hassell, Xiao-yu Zheng, Anastasia S. Lambrou, Rebecca Kondor, Duncan MacCannell, Natalie J. Thornburg, Joseph Miller, Dave Wentworth, Benjamin J. Silk

**Affiliations:** ^1^National Center for Immunization and Respiratory Diseases, CDC; ^2^Center for Preparedness and Response, CDC; ^3^National Center for Emerging and Zoonotic Infectious Diseases, CDC; ^4^Epidemic Intelligence Service, CDC.

Monitoring emerging SARS-CoV-2 lineages and their epidemiologic characteristics helps to inform public health decisions regarding vaccine policy, the use of therapeutics, and health care capacity. When the SARS-CoV-2 Alpha variant emerged in late 2020, a spike gene (*S*-gene) deletion (Δ69–70) in the N-terminal region, which might compensate for immune escape mutations that impair infectivity (*1*), resulted in reduced or failed *S*-gene target amplification in certain multitarget reverse transcription–polymerase chain reaction (RT-PCR) assays, a pattern referred to as *S*-gene target failure (SGTF) (*2*). The predominant U.S. SARS-CoV-2 lineages have generally alternated between SGTF and *S*-gene target presence (SGTP), which alongside genomic sequencing, has facilitated early monitoring of emerging variants. During a period when Omicron BA.5–related sublineages (which exhibit SGTF) predominated, an XBB.1.5 sublineage with SGTP has rapidly expanded in the northeastern United States and other regions.

As part of the Increasing Community Access to Testing (ICATT) program,* specimens collected at a national pharmacy chain were tested at a commercial laboratory that exclusively used the TaqPath COVID-19 Combo Kit (ThermoFisher Scientific) (*3*). Real-time RT-PCR cycle threshold (Ct) results for three gene targets (*S*, *N*, and *ORF1ab*) were reported to U.S. Department of Health and Human Services (HHS) Protect^†^ during November 1, 2021–January 14, 2023. The proportion of SGTF or SGTP^§^ (*2*) results was calculated weekly at the national and HHS regional levels^¶^; SGTF data were reported on a public dashboard.** CDC also collects genomic sequencing data from the National SARS-CoV-2 Strain Surveillance program,^††^ contracted commercial laboratories, and partners that label sequencing results in public repositories as baseline surveillance (*4*). Sequencing data are used to calculate variant proportions, which are published weekly on CDC’s COVID Data Tracker.^§§^ Genomic sequencing results lag 2–3 weeks behind specimen collection, which necessitates nowcasting estimates (*4*) for the most recent 3 weeks (December 25, 2022–January 14, 2023). Geographic representativeness and median interval from specimen collection to result were calculated for both data sources. Trends were assessed in SGTP proportions, variant proportions, and nowcast estimates; all were weighted to represent RT-PCR–positive specimens by state (*4*). Genomic sequencing results, including for a random sample of ICATT specimens, were assessed by SGTF/SGTP status. This activity was conducted consistent with applicable federal law and CDC policy.^¶¶^

During November 1, 2021–December 24, 2022, national weekly SGTF and SGTP results ranged from 3,104 to 83,805 (median = 102; IQR = 327 per jurisdiction***) and genomic sequencing results ranged from 6,313 to 69,280 (median = 195; IQR = 460 per jurisdiction^†††^). During December 25, 2022–January 14, 2023, the national weekly average number of SGTF/SGTP and sequencing results were 5,005 and 847, respectively. After specimen collection, SGTF/SGTP results were available sooner (median = 2 days; IQR = 1) than were genomic sequencing results (median = 16 days; IQR = 10).

Trends in SGTP proportions aligned with genomic sequencing results classified by SGTF and SGTP (Figure). For the week ending December 24, 2022, the latest week that weighted variant proportions were available from genomic sequencing, SGTP lineages accounted for 21.5% (XBB.1.5 = 11.8%; XBB = 4.4%; other BA.2-related sequences = 5.3%) of genomic sequences, while the weighted SGTP estimate from ICATT was 20% (95% CI = 18%–23%). For the week ending January 14, 2023, the SGTP estimate from ICATT was 40% (95% CI = 36%–45%); SGTP lineages from sequencing comprised 50.6% (XBB.1.5 = 43.0%; XBB = 3.9%; other BA.2-related sequences=3.7%) of lineages in the nowcast projections reported on January 13, 2023 and 45.5% (XBB.1.5 = 37.2%; XBB = 4.0%; other BA.2-related sequences=4.3%) of lineages in revised nowcast projections for the same week, subsequently reported on January 20, 2023%.^§§§ ^SGTP accounted for >50% of specimens in HHS regions 1–3 and >20% in all other regions, except Region 10, where the estimated precision was low (Supplementary Figure; https://stacks.cdc.gov/view/cdc/123810). Among genomic sequences from ICATT specimens collected through January 2, 2023, 412 (99%) of 415 XBB-related sequences exhibited SGTP; among those collected during December 1, 2022–January 2, 2023, 294 (59%) of 495 SGTP specimens were XBB-related lineages.

**FIGURE:**
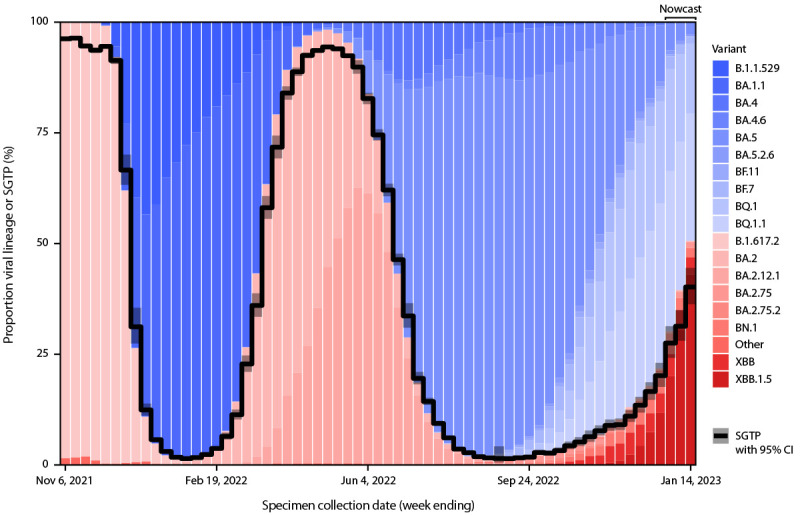
Trends in estimated proportions of SARS-CoV-2 reverse transcription–polymerase chain reaction test results with *S*-gene target presence and variant proportions and nowcast projections from genomic surveillance classified by *S*-gene target presence or *S*-gene target failure* — United States, November 1, 2021–January 14, 2023 **Abbreviations:** *S*-gene = spike gene; SGTF = *S*-gene target failure; SGTP = *S*-gene target presence. * Estimates of variant proportions and nowcast projections (for the most recent 3 weeks) are shown. The Delta (B.1.617.2) variant exhibited SGTP; the Omicron (B.1.1.529) variant and BA.1.1 sublineage exhibited SGTF; the Omicron BA.2 and BA.2.12.1 sublineages exhibited SGTP; Omicron BA.4 and BA.5 sublineages (which have the same spike sequence), BA.4-related (BA.4.6) and BA.5-related sublineages (BA.5.2.6, BQ.1, BQ.1.1, BF.7, and BF.11) exhibited SGTF; and BA.2-related sublineages (BA.2.75, BA.2.75.2, BN.1, XBB, and XBB.1.5) exhibited SGTP. The spike deletion (Δ69-70) that results in SGTF is not 100% penetrant in a lineage; SGTF/SGTP classification was made based on a 50% threshold. Most BA.2-related sublineages exhibit >99% SGTP.

SGTF/SGTP monitoring relies on diagnostic RT-PCR, which is less expensive, permits higher throughput, and faster turnaround of results than sequencing. Using SGTF/SGTP for early studies of emerging variants obviates the need to wait for sequencing results or >50% variant predominance. Limitations are that SGTF/SGTP monitoring is assay-dependent; presumes SARS-CoV-2 lineage classification, requiring further validation by genomic surveillance; cannot discriminate mutations beyond Δ69–70 (i.e., BA.2-related sequences or even between BA.4 and BA.5); and relies on continued changing SGTF/SGTP patterns compared with predominant lineages. SARS-CoV-2 sequencing remains the standard for genomic surveillance because it allows definitive classification of viral lineages and identification of emerging strains for further characterization.

When early nowcast estimates of rapidly emerging variants lacked precision and geographic resolution because of lags in genomic sequencing results, SGTF/SGTP estimates were used as complementary data by CDC and the SARS-CoV-2 Interagency Group to support guidance on the use of monoclonal antibody therapies.^¶¶¶^ SGTF/SGTP data were also used as proxy markers in several early studies of vaccine effectiveness and severity of emerging variants (*3*,*5*,*6*). Continued monitoring of SGTF/SGTP patterns will likely serve as a useful complement to genomic surveillance of SARS-CoV-2 lineages.

## Footnotes

*
https://www.cdc.gov/icatt/AboutICATT.html

† CDC’s HHS Protect is a secure decision-making and operations platform for the whole-of-government response to the COVID-19 pandemic and serves to collect, integrate, and share COVID-19 data across federal agencies and with state, local, territorial, and tribal partners. https://www.cdc.gov/ncezid/hhs-protect/index.html

§ Specimens with missing Ct values for *N* or *ORF1ab* were excluded. SGTF was defined as a SARS-CoV-2-positive specimen with amplification of the *N* and *ORF1ab* genes along with either a failure or reduced amplification of the *S* gene (*S* Ct value >4 cycles from the average of *N* and *ORF1ab* Ct values). SARS-CoV-2–positive specimens that were not SGTF were considered SGTP.

¶
https://www.hhs.gov/about/agencies/iea/regional-offices/index.html

** At the time of publication, the dashboard is not showing the SGTF proportions, but this functionality is expected to return in the near future. https://www.walgreens.com/businesssolutions/covid-19-index.jsp

††
https://www.cdc.gov/coronavirus/2019-ncov/variants/cdc-role-surveillance.html

§§
https://covid.cdc.gov/covid-data-tracker/#variant-proportions

¶¶ 45 C.F.R. part 46.102(l)(2), 21 C.F.R. part 56; 42 U.S.C. Sect.241(d); 5 U.S.C.0 Sect.552a; 44 U.S.C. Sect. 3501 et seq.

*** Forty-seven states, the District of Columbia, and Puerto Rico.

††† Fifty states, the District of Columbia, and five territories (American Samoa, Guam, Northern Mariana Islands, Puerto Rico, and the U.S. Virgin Islands).

¶¶¶
https://www.cdc.gov/coronavirus/2019-ncov/variants/variant-classifications.html
